# Targeting Cognitive Impairment in Multiple Sclerosis—The Road toward an Imaging-based Biomarker

**DOI:** 10.3389/fnins.2017.00380

**Published:** 2017-06-30

**Authors:** Jeroen Van Schependom, Guy Nagels

**Affiliations:** ^1^Center for Neurosciences, Universitair Ziekenhuis Brussel, Vrije Universiteit BrusselBrussels, Belgium; ^2^Radiology, Universitair Ziekenhuis Brussel, Vrije Universiteit BrusselBrussels, Belgium; ^3^Neurology, National MS Center MelsbroekMelsbroek, Belgium

**Keywords:** cognitive impairment, multiple sclerosis, biomarker, MRI and fMRI, neurophysiology

## Abstract

Multiple Sclerosis (MS) is a neuro-degenerative and -inflammatory disease leading to physical and cognitive impairment, pathological fatigue and depression, and affecting patients' quality of life and employment status. The combination of inflammation, demyelination, and neurodegeneration leads to the emergence of MS lesions, reduced white and gray matter brain volumes, a reduced conduction velocity and microstructural changes in the so-called Normal Appearing White Matter (NAWM). Currently, there are very limited options to treat cognitive impairment and its origin is only poorly understood. Therefore, several studies have attempted to relate clinical scores with features calculated either using T1- and/or FLAIR weighted MR images or using neurophysiology. The aim of those studies is not only to provide an improved understanding of the processes that underlie the different symptoms, but also to develop a biomarker—sensitive to therapy induced change—that could be used to speed up therapeutic developments (e.g., cognitive training/drug discovery/…). Here, we provide an overview of studies that have established relationships between either neuro-anatomical or neurophysiological measures and cognitive outcome scores. We discuss different avenues that may help to improve the prediction of cognitive impairment, and how well we can expect them to predict cognitive scores.

## Introduction

Cognitive impairment has been estimated to affect 1 out of every 2 multiple sclerosis (MS) patients (Rao et al., 1991) and affects different domains, most commonly information processing speed, working memory, long-term memory, attention, and executive functions (Langdon, 2011). In contrast to physical disability, which can be easily monitored with the Expanded Disability Status Scale (EDSS, Kurtzke, 1983), cognitive impairment is difficult to assess as (1) neuropsychological evaluation demands time and (2) test results can be influenced by practice-effects, i.e., an improvement of test scores even when the disease is stable as the patients get practiced in the specific tests.

Several attempts have been made to address these issues in order to facilitate and improve the reliability of cognitive follow-up. The Minimal Assessment of cognitive functioning in MS (MACFIMS, Benedict et al., 2002) and the Brief International Cognitive Assessment of MS (BICAMS, Langdon et al., 2012) have been developed in order to allow a less time-demanding cognitive assessment. Furthermore, even a single 5-min test can assess a patient's cognitive status with a sensitivity of up to 0.9 at a specificity of about 0.6 (Van Schependom et al., 2014a). Although, the practice effect can be partially mitigated by using alternate versions of cognitive tests, patients can still learn certain strategies limiting the potential of these batteries to detect changes in clinical trials.

As part of the clinical follow-up, MS patients regularly undergo an MRI scan allowing the radiologist/neurologist to assess the number and volume of T1-hypointense, T2-hyperintense, and Gadolinium enhancing lesions. While the automated interpretation of MR images has not only led to a more reliable quantification of lesions (Jain et al., 2015), it provides objective insight in the brain's atrophy rate, which is significantly faster in MS than in healthy controls (De Stefano et al., 2015).

Despite improvements in the quantification of MR images, only a limited correlation is observed between the radiological findings and a patient's actual physical or cognitive disability. This lack of correlation is well-known and is known as the clinico-radiological paradox, which can be caused by a multitude of factors, amongst which: (1) Neglect of damage to the spine when assessing the correlation between brain lesion load and physical disability; (2) The quality of clinical ratings; (3) Differences in cognitive reserve defined as *differences in cognitive processes as a function of lifetime intellectual activities and other environmental factors that explain differential susceptibility to functional impairment in the presence of pathology or other neurologic insult* (Barulli and Stern, 2013), for which intelligence quotient and education level are often taken as indicators (Martins Da Silva et al., 2015); and (4) The assumption that white and gray matter appearing normal on T1 and T2 weighted images are unaffected by the disease process (Barkhof, 2002). Although, the use of diffusion tensor imaging has shown that both normal appearing white and gray matter (normal with respect to their appearance on T1 and T2 weighted MRI, NAWM/NAGM) are affected, their inclusion does not solve the clinico-radiological paradox (Hawellek et al., 2011; Moll et al., 2011).

Apart from the structural damage, neurophysiological changes have been described. Already in 2000, Leocani et al. showed a reduced alpha power and an increase of power at lower frequencies in 40–80% of the MS patients (Leocani et al., 2000). As neurophysiological functioning is not only influenced by structure but also by more widespread changes that might be too subtle to be picked up with conventional MR imaging, it might help to reduce the clinico-radiological paradox.

Given the prevalence of cognitive impairment in MS, the difficulties in cognitive assessment and the lack of disease modifying therapies targeting cognition, we aim at providing an overview of possible roads toward a biomarker for cognition in MS based on neuroanatomical and neurophysiological features acquired through MRI or magneto-/electroencephalography (M/EEG). A biomarker that is more objective and reliable than standard neuropsychological tests, easy to acquire and sensitive to interventions could substantially improve the follow-up and therapeutic development.

## Neuro-anatomy

In multiple sclerosis, MR imaging has provided a unique way of assessing the disease activity in the patient's brain *in vivo*. It has allowed identifying hypo-intense lesions on T1-weighted images, hyper-intense lesions on T2-weighted images (Li et al., 2003) and active breaches of the blood-brain-barrier using T1-weighted images after the administration of Gadolinium as contrast-enhancer (Soon et al., 2007). Whereas, T1-hypointense lesions indicate axonal loss, T2 hyperintense lesions are known to be sensitive yet unspecific markers of disease activity (van Waesberghe et al., 1999). As such, MR images have provided a way of assessing the disease activity and are increasingly being used as secondary outcome in pivotal clinical trials (Cohen et al., 2012).

However, despite the easy interpretability and despite the inclusion of MR imaging parameters in the revised 2010 McDonald criteria (Polman et al., 2011) for the diagnosis of MS, the relationship between the parameters extracted from MR images and clinical disability, expressed in EDSS or cognitive scores, remains surprisingly low.

One explanation for this clinico-radiological paradox may be the use of univariate linear techniques, whereas the relationship between MRI covariates and clinical covariates does not necessarily need to be linear. In Hackmack et al. (2012), the authors argued that using canonical correlation analysis and a searchlight procedure, they obtained correlations of up to 80% using standard MR images. Yet, it is important to note that many of the areas that allowed to predict the clinical status involved the periventricular white matter, a region that is difficult to coregister to a template. As such, we should make sure to understand what features drive more advanced techniques, especially when extending toward machine learning.

### Atrophy

MS-related cognitive impairment has been associated with both cortical (Benedict, 2002; Benedict et al., 2005; Morgen et al., 2006) and subcortical (Houtchens et al., 2007; Sicotte et al., 2008; Batista et al., 2012; Damjanovic et al., 2016; Preziosa et al., 2016; Rocca et al., 2016) gray matter atrophy and cortical lesions (Calabrese et al., 2009) explaining between 20 and 60% of the variance of a variety of cognitive tests assessing the most commonly affected cognitive domains using multilinear models.

The relationship between cortical atrophy and cognition should not come as a surprise, given that neuronal density, neuronal size, and axonal density all significantly predicted gray matter volume in 45 tissue blocks in a post-mortem study (Popescu et al., 2015). A more extensive review on this topic can be found in Filippi (2015).

### Microstructural integrity

Apart from brain atrophy, MS leads to demyelination entailing a reduced structural connectivity. Normal appearing white matter typically shows decreased fractional anisotropy (FA), increased mean diffusivity (MD), and radial diffusivity (RD) demonstrating that the white matter appearing normal on T1 and T2 weighted MR images is likely to be affected by the MS pathology (Vrenken et al., 2006; Roosendaal et al., 2009; Hawellek et al., 2011; Moll et al., 2011).

Apart from general reductions/increases in diffusion parameters, several studies have found microstructural abnormalities in specific tracts like the fornix (Roosendaal et al., 2009; Kern et al., 2012), the cingulum (Mesaros et al., 2012), and the uncinate fasciculus (Fink et al., 2010). Furthermore, several of these changes correlate with cognitive impairment: e.g., a reduced information processing speed was associated with reduced FA in the corpus callosum (Roosendaal et al., 2009) and higher FA in the fornix was related to better memory results (Kern et al., 2012). Yet, importantly, Mesaros et al. found that lesional damage (assessed by FA/MD) along cognitive related tracts (especially the cingulum) outperformed diffusion abnormalities in NAWM when discriminating cognitively preserved and impaired MS patients (Mesaros et al., 2012) on a variety of neuropsychological tests.

Furthermore, the addition of these measures to multilinear models did not result in a substantial improvement of the prediction of general cognitive impairment (*R*^2^ = 0.2–0.5) and does not seem to solve the clinico-radiological paradox (Daams et al., 2015; Preziosa et al., 2016). One explanation for this result could be that the interpretation of abnormal diffusion parameters is not straightforward. While decreased FA and increased MD tend to point to increased diffusion and thus a reduced fiber integrity, both increased/decreased FA/MD may indicate pathology-induced changes depending on the brain region and the underlying cellular structure (Soares et al., 2013). Finally, the magnetization transfer ratio, a measure related to microglia activation and—only in close proximity of lesions—to axonal degeneration (Moll et al., 2011), has been shown to be altered before the onset of clinical symptoms (Iannucci et al., 2000).

## Neurophysiology

As functional connectivity (FC) is not only determined by the underlying structural connectivity matrix (Honey et al., 2009), it may provide additional and independent information on a patient's cognitive status and is therefore an important candidate biomarker for cognitive impairment in MS.

### Functional MRI

Based on the observation of the additional recruitment of adjacent brain areas during tasks in cognitively preserved MS patients as opposed to smaller activations in cognitively impaired MS patients (Staffen et al., 2002; Sweet et al., 2006), it has been suggested that the brain tries to compensate the reduced local processing power by recruiting adjacent areas leading to both increased activation and connectivity (Schoonheim et al., 2015).

However, recently, both increases (Hawellek et al., 2011; Faivre et al., 2012; Zhou et al., 2014) and decreases (Bonavita et al., 2011; Cruz-Gómez et al., 2013; Louapre et al., 2014) in resting-state FC of the default-mode network have been associated with cognitive impairment in MS. More specifically, Hawellek et al. observed an association between increased connectivity and impaired cognitive functioning and suggested that the widespread white-matter damage precludes the brain from easily switching between different states resulting in an increased FC (Hawellek et al., 2011).

These contradicting results have led Schoonheim et al. to doubt the “compensation” hypothesis, proposing that this increased activation may also be interpreted as a maladaptive response of the brain following e.g., disinhibition or even an unrelated side-effect of the accumulating structural damage (Schoonheim et al., 2015).

A closer look at the MS cohorts on which these contradicting results are based, shows that studies observing a higher FC in MS and positive correlations between cognitive impairment and functional connectivity included patients in the very early stage of the disease (mean disease duration: 2, 1.1, and 2.8 years in Hawellek et al., 2011; Faivre et al., 2012; Zhou et al., 2014) as compared to studies observing lower FC and negative correlations (mean disease duration of 5.5, 4.5, and 11 years in Bonavita et al., 2011; Cruz-Gómez et al., 2013; Louapre et al., 2014, respectively). Therefore, we suggest that increased FC may be related with increased cognitive impairment in the very early stage of the disease but with increased cognitive abilities in later disease stages.

### Magneto-/electroencephalography

All previously mentioned neurophysiological results are based on resting state functional MRI, which offers a high spatial resolution but does not capture the brain's rich temporal dynamics. Unfortunately, only few studies have assessed cognition in MS using resting-state assessed by electrophysiology (EEG) or magnetoencephalography (MEG).

The studies that have used rest EEG/MEG to assess MS patients, have found an increase in power density at low frequencies (delta, 2–4 Hz) and a decrease of power in the alpha band (Leocani et al., 2000; Babiloni et al., 2016), which allowed to distinguish between relapsing remitting and secondary progressive MS patients (Babiloni et al., 2016). Furthermore, Van der Meer et al. found a decrease in upper-alpha power (10–12 Hz) and an increase in lower-alpha power (8–10 Hz), which may relate to the slowing of the alpha-peak in Alzheimer's dementia (Goossens et al., 2017). Functional connectivity studies have observed an increase in functional connectivity in the beta band [assessed by the phase lag index (Tewarie et al., 2013), and synchronization likelihood (Schoonheim et al., 2013)] and the functional connectivity in the beta band correlated with an overall cognitive score (Tewarie et al., 2013).

With respect to task-related EEG/MEG, the most commonly applied paradigm is the P300, a paradigm in which the subject is asked to pay attention to a specific stimulus within a series of similar but more frequently occurring stimuli. Although, reduced amplitudes and increased latencies have been consistently found when comparing MS subjects with healthy controls (Piras et al., 2003; Magnano et al., 2006; Leocani et al., 2010), the accuracy of various features in detecting cognitive impairment in MS is limited: Van Schependom et al. reported accuracies of about 70% using a variety of machine learning techniques and features (Van Schependom et al., 2013, 2014c) with results highly depending on the choice of connectivity measure (Van Schependom et al., 2014b).

## Future directions

Given that both MRI and functional connectivity can only explain a small fraction of the observed variance, different approaches may be interesting to pursue.

One alternative approach to assessing the local connectivity of different structures is assessing the whole brain as one network. Based on this weighted or unweighted network—for a discussion on which metrics have been successfully applied cf. the EEG/MEG section above—different parameters can be calculated using graph theory. The most commonly defined parameters are the average shortest path length (also called the “integration”), the clustering coefficient and the modularity. Path length and clustering coefficient are typically normalized with respect to the mean of those parameters obtained by randomly permuting the adjacency matrix. The ratio of the normalized path length and clustering coefficient is called the small-worldness.

Graph theory approaches seem to point in the direction of a more regular topology as evidenced by an increase in path length and clustering coefficient in the alpha-band (Schoonheim et al., 2013) in rest and in the theta and delta band during an auditory oddball task (Van Schependom et al., 2014b).

An alternative option would be not to analyse the brain in terms of a frequency-decomposition, but rather to assess the brain in a non-static way. With regards to EEG, microstates—states that are stable for around 100 ms—have been shown to be relevant to schizophrenia (Kindler et al., 2011) and recently Gschwind et al. have shown differences in microstate properties in MS. Specifically, they found fewer short duration microstates and more frequent long duration states for the two microstates that have been suggested to represent the sensorimotor and the visual network (Gschwind et al., 2016). This finding could confirm the hypothesis put forward by Hawellek et al. of an impaired switching as the underlying mechanism of increased functional connectivity (Hawellek et al., 2011). Importantly, Gschwind et al. did not observe any correlation between the temporal dynamics and the patients' cognitive scores. This approach could be extended to MEG, where Baker et al. showed the existence of stable states with lifetimes around 100–200 ms. As these states are defined in source space, they are easier to interpret (Baker et al., 2014; Vidaurre et al., 2016).

## Further considerations

Finally, we should be aware of the fact that the “golden truth” of a patient's cognitive status cannot be directly observed but needs to be probed by the assessment of standardized neuropsychological tests. These tests have inherent limitations, e.g., some patients may have been subjected to similar tests previously and are better prepared than others. Furthermore, the results obtained on these tests may be influenced by a patient's mood and fatigue level, two factors that may even be more difficult to assess than cognition. A final covariate that mostly cannot be taken into account is the influence of medication [e.g., the use of anti-cholinergics to control bladder problems also influences cognitive functioning (Kersten et al., 2013) or the use of anti-epileptica (Ortinski and Meador, 2004)].

Therefore, a perfect correlation will never be reached. In order to provide a rough quantification of the correlation that we would be able to obtain, we assume (1) a true underlying cognitive profile that follows a Gaussian distribution across the MS population, (2) a simulated measured cognitive profile by adding extra Gaussian noise, and (3) a cognitive biomarker which is similarly composed of the sum of the true underlying cognitive profile and some Gaussian noise.

As Pearson's correlation coefficient decreases with increasing standard deviation of the measured cognitive score and the biomarker, both of which are increased by the additive Gaussian noise, the maximal accuracy will decrease. As an example, we can use the values provided by Boringa et al. (2001) for the SDMT (mean = 52, standard deviation = 11). Assuming a standard deviation of 3 points on both measurements, the theoretical maximum for *R*^2^ is 0.86. The main limitation of this type of calculation is the assumption on the distribution of both the underlying cognitive profile and the noise caused by either imprecise cognitive batteries or imprecise biomarkers. Apart from its common use, there is no specific reason for which we have chosen a Gaussian distribution. However, the main point that we aim to convey in this paragraph, i.e., that we cannot expect an imaging biomarker to be perfect because the assessment of cognition is not expected to be perfect either, is independent from the specific distribution.

## Conclusion

In this perspective paper, we described—without providing an exhaustive review—the different imaging modalities (MRI/DTI/MTI/EEG/MEG) that have been applied with the aim of finding a correlate of cognitive impairment in MS. While the features deduced from different MRI modalities do not seem to overcome the clinico-radiological paradox, the generally increased interest in assessing whole-brain functional networks using EEG/MEG has found its way to MS. Although, it is more difficult to interpret changes in power at certain frequency bands or the “effectiveness” of a functional network than it is to interpret the changes in the volume of different brain structures, we feel that improved EEG/MEG features—whether or not in combination with MRI—may help to reduce the paradox and lead to assess cognitive functioning more objectively (without inter-rater variability) and therefore lead to improved patient-care.

## Author contributions

JVS and GN have both made substantial contributions to the design and critical revision of the manuscript, approve the final version, and agree to be accountable for all aspects of the work.

### Conflict of interest statement

The authors declare that the research was conducted in the absence of any commercial or financial relationships that could be construed as a potential conflict of interest.
